# Freeze-Frame Imaging of Dendritic Calcium Signals With TubuTag

**DOI:** 10.3389/fnmol.2021.635820

**Published:** 2021-03-04

**Authors:** Alberto Perez-Alvarez, Florian Huhn, Céline D. Dürst, Andreas Franzelin, Paul J. Lamothe-Molina, Thomas G. Oertner

**Affiliations:** ^1^Institute for Synaptic Physiology, Center for Molecular Neurobiology, University Medical Center Hamburg-Eppendorf, Hamburg, Germany; ^2^Rapp OptoElectronic GmbH, Wedel, Germany

**Keywords:** calcium imaging, dendritic integration, two-photon (2p), hippocampus, genetically encoded activity indicators

## Abstract

The extensive dendritic arbor of neurons is thought to be actively involved in the processing of information. Dendrites contain a rich diversity of ligand- and voltage-activated ion channels as well as metabotropic receptors. In addition, they are capable of releasing calcium from intracellular stores. Under specific conditions, large neurons produce calcium spikes that are locally restricted to a dendritic section. To investigate calcium signaling in dendrites, we introduce TubuTag, a genetically encoded ratiometric calcium sensor anchored to the cytoskeleton. TubuTag integrates cytoplasmic calcium signals by irreversible photoconversion from green to red fluorescence when illuminated with violet light. We used a custom two-photon microscope with a large field of view to image pyramidal neurons in CA1 at subcellular resolution. Photoconversion was strongest in the most distal parts of the apical dendrite, suggesting a gradient in the amplitude of dendritic calcium signals. As the read-out of fluorescence can be performed several hours after photoconversion, TubuTag will help investigating dendritic signal integration and calcium homeostasis in large populations of neurons.

## Introduction

When a cluster of excitatory synapses is simultaneously activated on a basal dendrite of a pyramidal neuron, the combined depolarization triggers a local NMDA spike (Schiller et al., 2000). In addition to NMDA receptors, voltage-gated calcium channels also contribute to local dendritic depolarization and calcium spikes (Losonczy and Magee, 2006; Remy and Spruston, 2007; Takahashi et al., 2020). These non-linear processes may endow dendrites with the capability to serve as computational subunits (Polsky et al., 2004) and gate the output of cortical neurons during perception of sensory stimuli (Takahashi et al., 2020). As the dendritic arbor of even a single cortical neuron spans several square millimeters and dendritic calcium spikes are stochastic, capturing these rare events with laser scanning microscopy is technically challenging. Most of our knowledge is based on painstaking patch-clamp recordings from individual dendrites or simulating synaptic activity by local glutamate uncaging (Antic et al., 2010; Major et al., 2013). Optical recording *in vivo* is possible in head-fixed animals, but typically limited to a single plane containing a few dendrites (Helmchen et al., 1999; Takahashi et al., 2016). Several approaches to speed up volumetric imaging have been demonstrated (Bouchard et al., 2015; Szalay et al., 2016; Yang and Yuste, 2017), but the fundamental trade-off between the desired high spatial resolution in a large volume of tissue and the temporal resolution required to capture brief calcium transients cannot be solved by ever-faster scanning mechanisms (Lecoq et al., 2019). Fast multiphoton imaging requires high laser power, and the resulting local heating can affect brain physiology (Schmidt and Oheim, 2020). Thus, to analyze dendritic calcium signals in thousands of neurons under physiological conditions, it would be very attractive to temporally separate the labeling event from the read-out.

To generate a lasting record of synaptic activity that can be read-out later, we previously developed SynTagMA (Perez-Alvarez et al., 2020), a synaptically targeted variant of CaMPARI-2 (Moeyaert et al., 2018). This calcium integrator photoconverts irreversibly from green to red if it is bound to calcium and simultaneously illuminated by violet light (Fosque et al., 2015; Moeyaert et al., 2018). We reasoned that immobilizing the calcium integrator by targeting it to the dendritic cytoskeleton would allow generating a similarly conserved record (freeze frame) of dendritic calcium events. Here we show that a fusion of α-tubulin with CaMPARI2 provides stable cytoskeletal anchoring and very low turnover, essential features to preserve information about subcellular calcium distributions for later read-out. Using hippocampal slice cultures virally transduced with TubuTag as a test system, we show that electrical stimulation of Schaffer collateral axons induces calcium gradients in the apical dendrites, the distal tips reaching the highest concentrations. In fixed tissue, the contrast between active and inactive neurons and dendrites was further enhanced by an antibody that recognizes only the red form of TubuTag.

## Results

**FIGURE 1:**
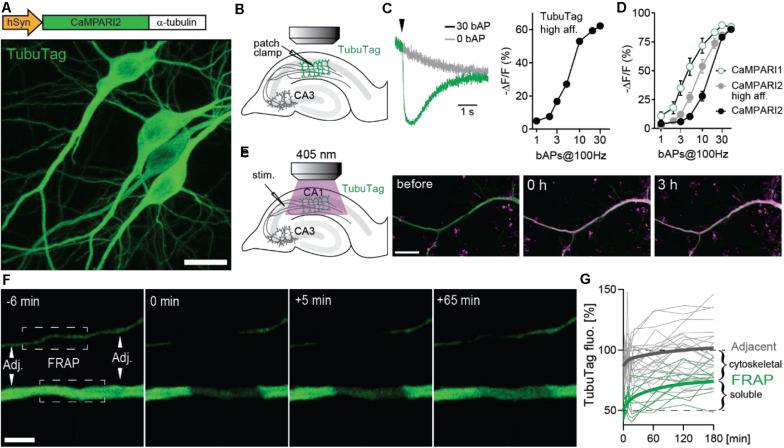
Design and performance of TubuTag. **(A)** TubuTag was created by fusing α-tubulin driven by the human synapsin-1 promoter to the calcium integrator CaMPARI2. Expression was tested by single-cell electroporation in CA1. Note absence of TubuTag from dendritic spines that are stabilized by f-actin. Scale bar: 20 μm. **(B)** Back-propagating action potentials (bAP) were induced in a TubuTag-expressing neuron while monitoring green fluorescence in the apical dendrite. **(C)** Dimming of high affinity TubuTag upon bAP generation through whole-cell patch clamp (2 neurons). Arrowhead indicates time of AP burst. Dimming increases with the number of bAPs. **(D)** Dimming of three soluble CaMPARI variants upon bAP induction. **(E)** High frequency synaptic stimulation via extracellular electrode (three 0.2 ms pulses, 100 Hz) in *stratum radiatum* followed by a long violet light pulse (405 nm, 14 mW/mm^2^, 500 ms duration, 1 s delay) led to strong photoconversion of TubuTag (10 pair repeats at 0.033 Hz). Converted TubuTag signal (red) in dendrites remained stable for over 3 h (2 neurons). Scale bar: 20 μm. **(F)** Turnover of TubuTag was assessed by fluorescence recovery after photobleaching (FRAP). Fluorescence at bleached (squares) and adjacent (arrowheads) dendritic areas was measured and followed over time. Scale bar: 5 μm. **(G)** Dendritic fluorescence normalized to F_0_ fitted by a double exponential for bleached (τ_fast_ = 8 min; τ_slow_ = 75 min, *n* = 12 dendrites, 6 neurons) and adjacent areas.

Our new sensor is based on the fluorescent protein mEos (Wiedenmann et al., 2004) that undergoes irreversible photoconversion from green to red when illuminated with violet light (405 nm). Previously, mEos had been fused to a calcium-dependent conformation switch (calmodulin-M13) to create integrating sensors that can only be photoconverted when bound to calcium (CaMPARI, CaMPARI2) (Fosque et al., 2015; Moeyaert et al., 2018). To generate TubuTag, we fused CaMPARI2 in-frame to α-tubulin. We also created a high affinity version based on CaMPARI2_F391W_L398V (Moeyaert et al., 2018). Expressed in hippocampal neurons by single cell electroporation, dendrites, and somata showed bright green fluorescence. Dendritic spines, which rarely contain microtubules and are stabilized by f-actin instead, were largely invisible (Figure 1A). As the green fluorescence of the CaMPARI moiety dims upon calcium binding, it can be used as an acute calcium sensor. To characterize the calcium sensitivity of TubuTag, we induced back-propagating action potentials (bAPs) by somatic current injections and simultaneously measured green fluorescence intensity in the apical dendrite by two-photon microscopy (Figure 1B). Interleaved trials with no stimulation (0 APs) allowed distinguishing bleaching and wash-out of TubuTag from calcium-induced dimming (Figure 1C). The dimming response increased with the number of bAPs in a 100 Hz burst, following a sigmoidal curve in a log dose-response plot. We found that our tubulin fusion constructs followed the binding curves of the parent CaMPARI variants (Figure 1D) up to 10 bAPs, but reached saturation slightly earlier (−62% ΔF/F after 30 bAPs). For this study, we used the lowest affinity TubuTag (with CaMPARI2) to minimize baseline photoconversion in inactive cells. CaMPARI2 is also much brighter and shows more efficient photoconversion (larger dynamic range) than CaMPARI1 (Moeyaert et al., 2018).

Illumination with long violet light (405 nm) pulses during extracellular high-frequency stimulation of CA3 axons induced strong photoconversion of CA1 neurons expressing TubuTag (Figure 1E). The red label showed no sign of decay after 3 h, indicating irreversible photoconversion and slow protein turnover. Therefore, we expect red/green ratio differences between active and inactive neurons to be stable for several hours. To determine the fraction of TubuTag integrated in stable microtubules vs. soluble TubuTag monomers, we measured fluorescence recovery after photobleaching (FRAP, Figure 1F). After 3 h, ∼50% of the bleached fluorescence had recovered (Figure 1G), consistent with the slow turnover of microtubules in neurons (Kahn et al., 2018). Adjacent regions showed a small and transient dip in fluorescence intensity, consistent with diffusional exchange of bleached and unbleached monomers. Thus, if subcellular resolution is the goal of the experiment and 5% loss of contrast is acceptable, the red/green ratio (R/G) should be determined within ∼40 min after photoconversion.

**FIGURE 2:**
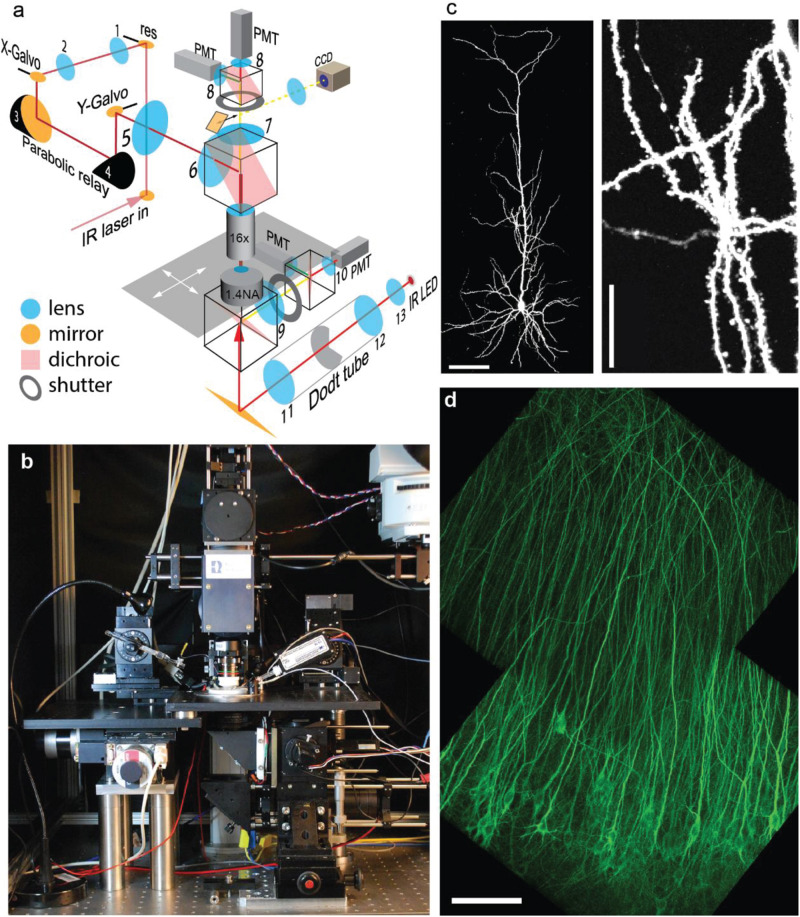
Design and performance of the Rapid3D microscope. **(a)** The pulsed IR laser is scanned via resonant (res) and galvanometric scan mirrors (*X*-Galvo, *Y*-Galvo) through a scan lens – tube lens assembly (5–6) and a 25 × 1.0 NA objective. The first relay (1–2) provides a magnification factor *M* = 1.3, the parabolic relay (3–4) has *M* = 1, the scan lens – tube lens assembly (5–6) provides *M* = 4.5. Fluorescence is detected by 4 photomultiplier tubes (PMT) through red and green band pass filters. A substage IR LED provides Dodt contrast illumination (CCD camera) for patch clamp experiments. **(b)** Frontal view of the Rapid3D microscope. **(c)** Image (maximum intensity projection) of a single CA1 pyramidal neuron expressing a cytoplasmic red fluorescent protein (tdimer2). Dendritic spines and axonal boutons are well resolved. Scale bars: 100 μm (left), 20 μm (right). **(d)** Virally transfected CA1 pyramidal cells expressing TubuTag. Note apparent absence of dendritic spines as they rarely contain microtubules. Scale bar: 100 μm.

To image large populations of neurons at high resolution, low-magnification high-NA water immersion objectives are available from all major microscope manufacturers. However, it is challenging to integrate them into commercial two-photon microscopes as they require a large diameter laser beam scanned at large angles with minimal wave front error. In addition, the detection pathway has to conserve the etendue of the objective (or condenser, respectively) in order to detect all emitted photons that enter the optical system. We custom-built a two-photon microscope designed to meet the following requirements: (1) 800 × 800 μm field of view (FoV); (2) efficient detection of green and red fluorescence, simultaneously through the objective and through the condenser; (3) resonant-galvo-galvo scanning for fast frame rates; (4) protection of photomultiplier tubes (PMTs) during photoconversion (405 nm light). In our system, the scan lens – tube lens assembly provides 4.3 × magnification and is horizontally oriented, allowing for a compact detection assembly directly on top of the objective (Figures 2a,b). Below the stage, a second optical system detects fluorescence through a 1.4 NA oil immersion condenser. For patch-clamp experiments, a CCD camera is coupled in by a sliding 45° mirror and the condenser provides Dodt contrast illumination. During photoconversion, both light detection pathways are protected by electronic shutters with 45 mm free aperture (NS45B, Uniblitz, United States).

To maintain the brain tissue at 37°C throughout the experiment, the temperature of the perfusate was controlled by an in-line heater and the oil-immersion condenser was heated to 33°C by a ring of Peltier elements, with the cool side in contact with the PMT assembly. A magnetic holder allows switching objectives with different working distances for different types of experiments. For most experiments, we used a Nikon 16 × 0.8 NA objective which provides a good compromise between FoV, optical resolution (Figures 2c,d) and 45-degree approach angle for patch-clamp or stimulation electrodes.

**FIGURE 3:**
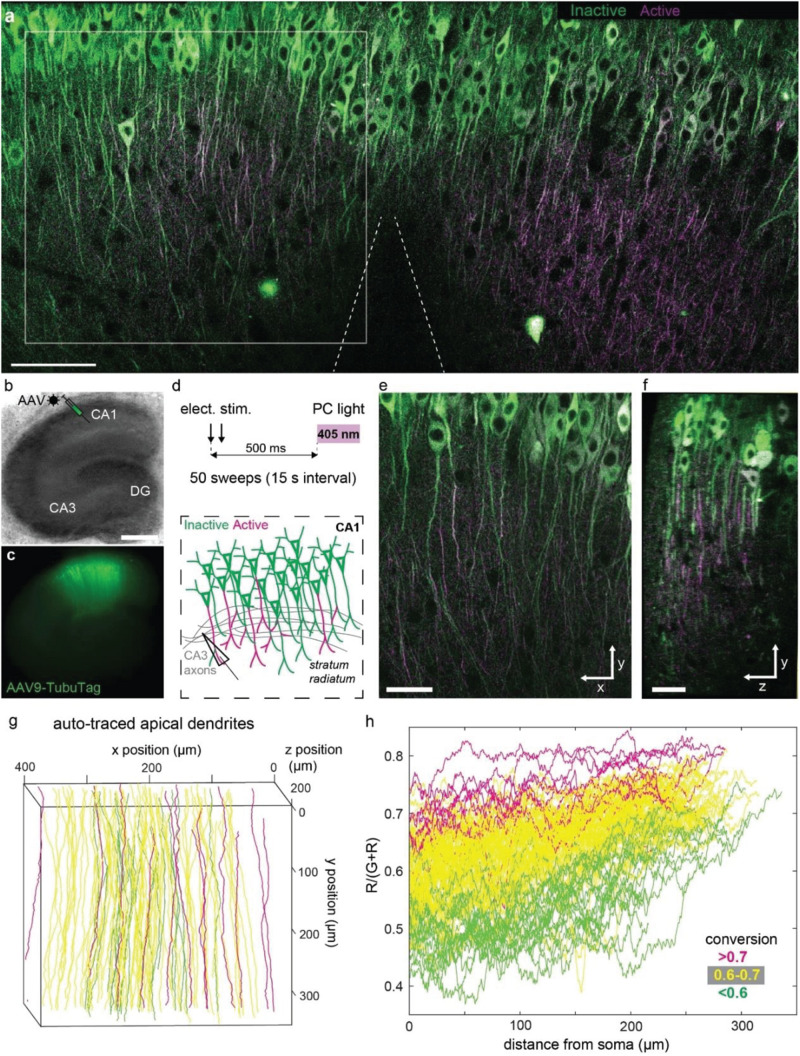
Photoconversion of TubuTag by extracellular stimulation paired with violet light. **(a)** Two-photon image (single plane) of CA1 after pairing extracellular stimulation with violet light. Scale bar: 100 μm. **(b)** Rat hippocampal slice culture microinjected with AAV9-syn-TubuTag in the CA1 region. Scale bar: 500 μm. **(c)** Epifluorescence image of the transduced culture. **(d)** To account for the slow conformational change of TubuTag upon Ca^2+^ binding, photoconversion light (405 nm, 11 mW/mm^2^, 200 ms duration) was delayed relative to the electrical stimulation of Schaffer collateral axons. This stimulation was repeated 50 times. **(e)** Detail of **(a)** showing strong differences in photoconversion between neighboring dendrites. Scale bar: 50 μm. **(f)** Side view of the volume shown in **(e)**. Scale bar: 30 μm. Note that photoconversion is not restricted to superficial dendrites (left), but independent of depth. **(g)** Automatic tracing of apical dendrites, color coded according to their average conversion [R/(G + R)]. Traced region corresponds to gray square in **(a)**, but extends further in depth. **(h)** Photoconversion profiles of the individual dendrites shown in **(f)**, color coded according to their average conversion. Note trend to stronger photoconversion toward the distal tips (right).

In order to express our sensor in a population of neurons, we packaged TubuTag into an AAV9 vector and injected it into the CA1 region of hippocampal organotypic slices using a Picospritzer (Figures 3a–c). Schaffer collateral axons were stimulated with an extracellular electrode in *stratum radiatum* (paired pulses at 20 Hz), shortly followed by a short violet light pulse delivered through the imaging objective (Figure 3d). After repeating this protocol 50 times, many dendrites in the vicinity of the electrode turned red while somata showed little photoconversion (Figure 3a). Strongly converted dendrites could be found next to non-converted dendrites, suggesting that not all neurons produced dendritic calcium spikes (Figure 3e). Conversion efficiency was not different in superficial vs. deep dendrites, indicating good penetration of the 405 nm photoconversion light (Figure 3f). The high transduction efficiency of AAV9 allowed us to monitor compartmentalized calcium signals at full density in a large volume fraction of CA1 in a single experiment. We used a tracking algorithm (see the section “Materials and Methods”) to trace apical dendrites from the soma to the distal tips (Figure 3g). Not surprisingly, the density of strongly photoconverted dendrites increased toward the stimulation electrode. Evaluating photoconversion along individual dendrites revealed that distal tips were more strongly converted, even in neurons where little or no photoconversion was detected in the proximal apical dendrite (Figure 3h). The large surface-to-volume ratio of thin distal dendrites would be expected to favor large amplitude calcium signals. As thin dendrites are much smaller than the point-spread function of a two-photon microscope, the amplitude of these signals may have been systematically underestimated in acute calcium imaging experiments with single wavelength indicators.

**FIGURE 4:**
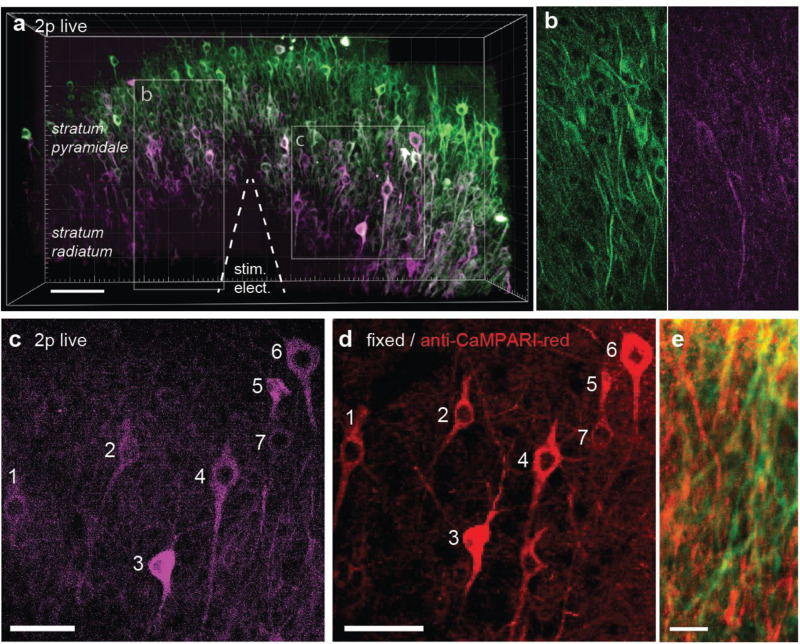
TubuTag immunodetection. **(a)** Three-dimensional display of CA1 area of hippocampal slice virally transfected with TubuTag after photoconversion (smoothed with Gaussian filter). Electrical stimulation at 37°C followed by long violet light illumination (405 nm, 11 mW/mm^2^, 500 ms duration, 1 s delay, 50 pair repeats at 0.1 Hz). Scale bar: 100 μm. **(b)** Detail (single optical plane, raw image) of green and red channel, showing photoconverted dendrites. **(c)** Detail of somatic layer (maximum intensity projection), showing photoconverted neurons (magenta). Scale bar: 50 μm. **(d)** Confocal image of same region as in **(b)** after fixation and staining against red (i.e., photoconverted) TubuTag. The far-red secondary antibody is displayed in red. Scale bar: 50 μm. **(e)** Detail of dendrites in stratum radiatum, overlay of endogenous green fluorescence and antibody-enhanced red fluorescence. Scale bar: 10 μm.

It would be attractive to induce TubuTag conversion in intact animals during behavior and image the frozen calcium signal at high resolution *ex vivo*. Chemical fixation, however, invariably leads to some loss of fluorescence, reducing image contrast. For *ex-vivo* detection of active dendrites, it would be advantageous to specifically enhance the red fluorescence by immunostaining. A specific antibody against the red form of CaMPARI has been developed (Moeyaert et al., 2018). As a proof of principle, we tested this antibody on slices where TubuTag-expressing neurons had been strongly stimulated and illuminated with violet light, resulting in layer-specific photoconversion across the whole CA1 region (Figures 4a,b). After fixation, we used the primary anti-CaMPARI-red antibody and a far-red secondary antibody (AF648) to selectively enhance the photoconverted signal. Anti-CaMPARI-red highlighted the same neurons we previously identified as converted, indicating excellent specificity for the photoconverted form of TubuTag (Figures 4c,d). At higher magnification, photoconverted dendrites could be identified by their high immunoreactivity (Figure 4e).

## Discussion

Calcium signals provide a unique window into neuronal physiology. From the release of neurotransmitter, induction of synaptic plasticity, complex spike bursts, to immediate early gene expression: all significant events in the life of a neuron are accompanied by calcium transients. Two-photon microscopy is a powerful tool to monitor such intracellular calcium signals at high resolution in intact brain tissue. Since fluorescence is sampled point-by-point, however, imaging of large and densely labeled volumes at the speed necessary to catch physiological calcium signals in subcellular detail is challenging. The calcium integrator CaMPARI irreversibly labels active neurons in a time window defined by violet illumination (Fosque et al., 2015; Moeyaert et al., 2018). This was a revolutionary concept, since it completely dissociated the labeling event from the optical recording of the results. Obviously, the speed of the microscope becomes much less important once the functional signal is frozen in time. Since CaMPARI is free to diffuse inside a neuron, subcellular gradients in photoconversion rapidly dissipate and the location of active inputs is lost. By fusing CaMPARI2 to tubulin, we immobilized the calcium reporter, providing a lasting record of high [Ca^2+^] areas across the dendritic arbor. To be able to interpret differential TubuTag photoconversion as [Ca^2+^] differences, it is important to ensure homogenous intensity of the photoconversion light across the brain region of interest.

For the previously published synaptically localized calcium integrator SynTagMA (Perez-Alvarez et al., 2020), we acquired separate image stacks to collect green and red fluorescence, exciting at 980 and 1040 nm, respectively. Green images had very little autofluorescence and were used to mask out non-synaptic fluorescence in the red channel. Due to chromatic aberration and imperfect alignment of the two laser lines, however, image registration was required before SynTagMA analysis. For TubuTag, we used a single wavelength (1,040 nm) used to excite both green and red species simultaneously. This simplified the analysis, as there was no need to register green and red images prior to analysis.

A topic of considerable interest is the possibility that synapses with synchronous activity are able to stabilize each other when situated close together, leading over time to a clustered input organization (Kleindienst et al., 2011; Iacaruso et al., 2017). In principle, an activity tag exclusively located at excitatory synapses like postSynTagMA (Perez-Alvarez et al., 2020) could be used to investigate the input organization of individual neurons, but photoconversion of a group of spines could also result from a local dendritic calcium event. Combining SynTagMA and TubuTag might be a way to resolve this ambivalence: red spines on a green section of dendrite would be incontrovertible evidence for synchronous synaptic activity. If the postsynaptic neuron spikes at high frequency, however, not only input sites, but also other parts of the dendrite would become photoconverted. For precise localization of active inputs, it may thus be useful to co-express TubuTag with a light-gated potassium or chloride channel (Wietek et al., 2015; Beck et al., 2018) to prevent action potential generation during the photoconversion period.

The most important feature of TubuTag is the hour-long preservation of subcellular [Ca^2+^] gradients in live tissue. Here we report the gradient along the long apical dendrite of CA1 pyramidal cells, but it would also be very interesting to investigate dendritic calcium events in interneurons, which are known to have a much higher calcium buffering capacity (Francavilla et al., 2019; Topolnik and Camiré, 2019). We envision that a head-mounted violet LED could be used to trigger photoconversion in intact animals, a situation where high-resolution imaging is very challenging. As we show, the signal-to-noise ratio in fixed tissue can be considerably improved by an antibody that recognizes only the red form of TubuTag. Compared to acute two-photon calcium imaging *in vivo*, which usually requires head fixation, TubuTag may provide a way to map cortical activity in freely moving animals.

## Materials and Methods

### Plasmid Construction

TubuTag is CaMPARI2 (Moeyaert et al., 2018) fused to human α-tubulin, high affinity TubuTag contains 2 point mutations in the CaMPARI2 domain, F391W and L398V. To generate high affinity TubuTag, we replaced mCherry from pcDNA3.1 mCherry-human-alpha-tubulin (a gift from Marina Mikhaylova) with the CaMPARI moiety from preSynTagMA (Perez-Alvarez et al., 2020; Addgene ID 119738) using *Hin*dIII and *Bsp*1407I restriction sites. To insert the CaMPARI-tubulin fusion construct into a pAAV backbone, we first replaced in the C-terminus the restriction site *Xho*I by *Hin*dIII using PCR. Subsequently, we used *Nhe*I and *Hin*dII to insert it into the pAAV backbone of pAAV-syn-ChR2ETTC (Berndt et al., 2011; Addgene ID 101361). To create TubuTag, we mutated residues W391 and V398 by overlap extension PCR to obtain a W391F_V398L variant (CaMPARI2). Briefly, we designed primers to amplify two DNA segments containing both the point mutations, namely between *Nhe*I and V398L, W391F and *Box*I. The resulting amplified segments with sizes 1304 bp and 728 bp were overlaid, resulting in a 1968 bp segment size that was inserted to replace the segment in TubuTag using restriction sites *Nhe*I and *Box*I. TubuTag (Addgene ID 164184) and high affinity TubuTag (Addgene ID 164193) are available at Addgene.org.

### Organotypic Hippocampal Slice Cultures

Hippocampal slice cultures from Wistar rats of either sex were prepared at postnatal day 4–7 as described (Gee et al., 2017). Briefly, rats were anesthetized with 80% CO_2_ 20% O_2_ and decapitated. Hippocampi were dissected in cold slice culture dissection medium containing (in mM): 248 sucrose, 26 NaHCO_3_, 10 glucose, 4 KCl, 5 MgCl_2_, 1 CaCl_2_, 2 kynurenic acid, and 0.001% phenol red. pH was 7.4, osmolarity 310–320 mOsm kg^–1^, and solution was saturated with 95% O_2_, 5% CO_2_. Tissue was cut into 400 μM thick sections on a tissue chopper and cultured at the medium/air interface on membranes (Millipore PICMORG50) at 37°C in 5% CO_2_. No antibiotics were added to the slice culture medium which was partially exchanged (60–70%) twice per week and contained (for 500 ml): 394 ml Minimal Essential Medium (Sigma, United States), 100 ml heat inactivated donor horse serum (Sigma, United States), 1 mM L-glutamine (Gibco, United States), 0.01 mg ml^–1^ insulin (Sigma, United States), 1.45 ml 5 M NaCl (Sigma, United States), 2 mM MgSO_4_ (Fluka, United States), 1.44 mM CaCl_2_ (Fluka, United States), 0.00125% ascorbic acid (Fluka, United States), 13 mM D-glucose (Fluka). Wistar rats were housed and bred at the University Medical Center Hamburg-Eppendorf. All procedures were performed in compliance with German law and according to the guidelines of Directive 2010/63/EU. Protocols were approved by the Behörde für Justiz und Verbraucherschutz (BJV) – Lebensmittelsicherheit und Veterinärwesen, of the City of Hamburg.

### Neuronal Transduction

CA1 neurons in rat organotypic hippocampal slice culture (DIV 17–21) were transfected by single-cell electroporation (Wiegert et al., 2017a). Thin-walled pipettes (∼10 MΩ) were filled with intracellular K-gluconate based solution into which plasmid DNA was diluted to 20 ng μl^–1^. Pipettes were positioned against neurons and DNA was ejected using an Axoporator 800A (Molecular Devices) with 50 hyperpolarizing pulses (−12 V, 0.5 ms) at 50 Hz. Experiments were conducted 3–5 days after electroporation.

For viral transduction of hippocampal slices, AAV2/9 viral vectors containing TubuTag variants under the control of the synapsin promoter was prepared at the UKE vector facility. Working in a laminar air flow hood, organotypic hippocampal slices were microinjected at DIV 7–11 (Wiegert et al., 2017b). A pulled glass pipette was backfilled with 1 μl of the viral vector and the tip inserted in the hippocampal CA1 area. A picospritzer (Parker, United States) coupled to the pipette was used to deliver 3–4 short (50 ms) low pressure puffs of viral vector into the tissue. Injected slices were taken back to the incubator and imaged in the two-photon microscope 15–21 days later.

### Two-Photon Imaging and Photoconversion

For fast two-photon imaging, we used a customized version of the Rapid3Dscope (Rapp OptoElectronic GmbH, Germany), a large field of view two-photon microscope equipped with relayed resonant-galvo-galvo scanners (RGG). The microscope is controlled by the open access software ScanImage 2017b (Vidrio, United States; Pologruto et al., 2003). To simultaneously excite both the green and red species of TubuTag, we used a Ti:Sapphire laser (Chameleon Ultra II, Coherent, United States) tuned to 1040 nm, controlled by a Pockels cell (Conoptics, United States) and an electronic shutter (Uniblitz, United States). Red and green fluorescence was detected through upper and lower detection path with one pair of photomultiplier tubes (PMTs, H7422P-40SEL, Hamamatsu, Japan) on each side. The upper detection is placed above the objective (CFI75 LWD, 16x, 0.8 NA, Nikon, Japan), using a short-pass dichroic mirror (Chroma ZT775sp-2p) and a secondary red/green splitter (Chroma T565lpxr, United States) with red and green band path emission filters (Chroma ET525/70m-2p, Chroma ET605/70m-2p, United States). The lower detection uses an oil immersion condenser (1.4 NA, Olympus, Japan) and equivalent red/green detection optics. With the 16 × 0.8 NA Nikon objective, axial resolution was 4.0 μm in the center of the image, 5.5 μm at a lateral distance of 212 μm and 7.1 μm at a lateral distance of 425 μm (FWHM of Gaussian fits to the axial intensity profile of 175 nm green fluorescent beads in agarose, excited at 980 nm).

For extracellular synaptic stimulation, a glass monopolar electrode filled with extracellular solution was placed in *stratum radiatum*. Paired 0.2 ms pulses (40 ms inter stimulus interval) were delivered using an ISO-Flex stimulator (A.M.P.I., Israel) at 0.1 Hz. Overview images were acquired, covering an area close to the electrode tip. The resonant-galvo-galvo scanner allowed z-stack acquisition covering a brain volume of 435 μm × 435 μm × 100 μm in 2 min at 1024 × 1024 pixel resolution and 1 μm z-steps. Homogenous photoconversion light (405 nm) was provided by coupling an LED light source (CoolLED pE4000, United Kingdom) via liquid light guide (3 mm dia.) into the microscope. The end of the liquid light guide, which homogenizes the Lambertian emission profile of the LED, was projected into the specimen plane of the microscope (critical illumination).

Electrical stimulation and 405 nm illumination were controlled by the open source software WaveSurfer^1^. Electronic shutters (NS45B, Uniblitz, United States) protected PMTs during photoconversion. Series of 2–3 images acquired to cover a mm-long brain area were registered using the pairwise stitching plugin from ImageJ (Preibisch et al., 2009). For suprathreshold stimulation and photoconversion, three 0.2 ms pulses at 100 Hz were applied with a glass monopolar electrode followed 1 s later by 500 ms violet light (14 mW/mm^2^). This pairing was repeated ten times at 0.033 Hz.

For calcium sensitivity calibration, CA1 neurons expressing TubuTag or CaMPARI variants or 3–5 days after electroporation were patched and brought into whole-cell configuration. Back-propagating action potentials (bAPs) were induced by injecting 1 nA pulses of 1 ms duration in current clamp. Fluorescence in the main apical dendrite was acquired at 500 Hz and changes from basal values were reported as ΔF/F (F-F_0_/F_0_). To depict photoconversion, red and green channels were merged.

### Automated Tracing of Apical Dendrites

We used the Fiji plugin ParticleTracker 2D (MOSAIC suite, Germany) to trace cross-sections of apical dendrites (“particles”) from the soma to their distal tips. Red and green fluorescence was added prior to particle detection to avoid bias for (or against) strongly converted dendrites. At bifurcations, the algorithm followed the brighter branch. Only long trajectories (>450 points) were considered for analysis. The coordinates of the tracked dendrites were exported to Matlab to extract red and green fluorescence intensities from the original stacks. Single-voxel values were smoothed along the dendritic track (41-point Gaussian kernel) to reduce the impact of shot noise. We then calculated R/(G + R) to normalize results between 0 (no conversion) and 1 (full conversion); actual values ranged from 0.4 to 0.8. We defined strongly converted dendrites as having a mean conversion ratio >0.7.

### Fluorescence Recovery After Photobleaching (FRAP)

To estimate the diffusion of TubuTag molecules, we took *z*-stacks of dendrites in *stratum radiatum* from neurons electroporated with TubuTag and we bleached central parts of dendrites, subsequently following the recovery of fluorescence over time. After obtaining a baseline of 1–2 images, we zoomed (4X) onto the dendrite and performed 10 *z*-stacks at twice the laser power to achieve ∼50% bleaching without signs of tissue damage. Images were subsequently taken at the original settings and magnification at different intervals for 180 min. For analysis of each dendrite, we used a macro written in Fiji (Schindelin et al., 2012) to quantify fluorescence changes over time (Moeyaert et al., 2018) in the bleached area and two immediately adjacent non-bleached areas. Kinetics of recovery after bleaching was fit using non-linear regression in Prism 8 (GraphPad, United States).

### Immunohistochemistry

After imaging and photoconversion in the 2p microscope, slices were fixed in a PBS solution containing 4% PFA for 30 min at room temperature (RT), washed and kept overnight in PBS at 4°C. Slices were then incubated in blocking buffer (1x PBS, 0.3% TritonX, 5% goat serum) for 2 h at RT. Next, sections were placed in the primary antibody (1:1,000, mouse Anti-CaMPARI-Red 4F6-1 clone, a gift from Eric Schreiter) carrier solution (PBS, 0.3% TritonX, 1% goat serum, 1% BSA) and incubated overnight at 4°C. After 3x washing with 1xPBS for 5 min, the sections were incubated for 2 h at RT in the secondary antibody (1:1,000, goat anti mouse Alexa Fluor 647, Life Technologies, United States) carrier solution (1x PBS, 0.3% TritonX, 5% goat serum). Sections were washed 3 × 10 min in 1x PBS, mounted on coverslips using Immu-Mount (Shandon, United States), and imaged with a confocal microscope (Olympus FV 1000) using a 20x oil immersion objective (UPLSAPO 20X NA 0.85). Two-channel images were obtained at 1024 × 1024 pixel resolution. Excitation/emission spectra and filters were selected using the automatic dye selection function of the Olympus FluoView software (Alexa 488 and Alexa 647).

## Data Availability Statement

The raw data supporting the conclusions of this article will be made available by the authors, without undue reservation.

## Ethics Statement

The animal study was reviewed and approved by the Behörde für Justiz und Verbraucherschutz (BJV) – Lebensmittelsicherheit und Veterinärwesen, of the City of Hamburg. All procedures were performed in compliance with German law and according to the guidelines of Directive 2010/63/EU.

## Author Contributions

AP-A, PJL-M, and AF conducted the experiments and analyzed the data. FH, AP-A, and CDD designed and built the microscope. TGO and AP-A conceived the experiments. AP-A wrote the first draft of the manuscript. All authors contributed to the final version.

## Conflict of Interest

FH is an employee of Rapp OptoElectronic GmbH, the company which designed and manufactured the prototype microscope. AP-A and CDD received support from Rapp OptoElectronic GmbH. The remaining authors declare that the research was conducted in the absence of any commercial or financial relationships that could be construed as a potential conflict of interest.

## Footnotes

1 https://wavesurfer.janelia.org/
